# ORGANIZATIONAL READINESS TO CHANGE AND NURSING HOME SAFETY: RESULTS FROM A NATIONAL SURVEY

**DOI:** 10.1093/geroni/igz038.2821

**Published:** 2019-11-08

**Authors:** Christine W Hartmann, Emma Quach, Shibei Zhao, Valerie Clark, Sarah McDannold, Pengsheng Ni, Lewis Kazis

**Affiliations:** 1 Bedford VA Medical Center, Bedford, Massachusetts, United States; 2 VA Center for Healthcare Organization and Implementation Research, Bedford, Massachusetts, United States; 3 Boston University School of Public Health, Boston, Massachusetts, United States

## Abstract

In nursing homes, safety climate (employee attitudes and beliefs about safety) is a key contributing factor to safety and a potential leverage point for improvement. Yet relatively little is known about how contextual factors such as organizational readiness to change affect safety climate. We sampled employees from 56 Department of Veterans Affairs (VA) Community Living Centers (CLCs—nursing homes) and conducted an anonymous, cross-sectional web-based survey using the previously validated CLC Employee Survey of Attitudes about Resident Safety (CESARS) and the Organizational Readiness to Change Assessment instrument. From hierarchical mixed random effects regression models, we calculated intraclass correlation coefficients (ICC) as the proportion of CLC-level variance over the sum of CLC-level plus residual variance. Each of the CESARS’ 7 safety climate domains was a dependent variable in separate models; employee- and CLC-level factors were independent variables. The survey had a 26% response rate; 1,397 respondents. Mean ORCA scores (1-5 scale, higher better) was 3.3. We began with models containing only employee-level variables. ICC values ranged from 2.34% to 9.85%, suggesting substantial variation in CESARS outcomes. As we dropped insignificant variables and added CLC-level variables to the models, the ICC decreased over 2% in six models, suggesting organizational-level variables accounted for substantial variability. The only independent variable with a significant effect in all 7 models was organizational-level: organizational readiness to change. Unlike many other organizational-level variables, organizational readiness to change is potentially amenable to low-cost interventions such as communication and teamwork interventions, providing viable opportunities to efficiently improve nursing home care.

